# Muscle-Bone Crosstalk in Chronic Obstructive Pulmonary Disease

**DOI:** 10.3389/fendo.2021.724911

**Published:** 2021-09-28

**Authors:** Lijiao Zhang, Yongchang Sun

**Affiliations:** Department of Respiratory and Critical Care Medicine, Peking University Third Hospital, Beijing, China

**Keywords:** COPD, sarcopenia, osteoporosis, myokines, osteokines, crosstalk

## Abstract

Sarcopenia and osteoporosis are common musculoskeletal comorbidities of chronic obstructive pulmonary disease (COPD) that seriously affect the quality of life and prognosis of the patient. In addition to spatially mechanical interactions, muscle and bone can also serve as endocrine organs by producing myokines and osteokines to regulate muscle and bone functions, respectively. As positive and negative regulators of skeletal muscles, the myokines irisin and myostatin not only promote/inhibit the differentiation and growth of skeletal muscles, but also regulate bone metabolism. Both irisin and myostatin have been shown to be dysregulated and associated with exercise and skeletal muscle dysfunction in COPD. During exercise, skeletal muscles produce a large amount of IL-6 which acts as a myokine, exerting at least two different conflicting functions depending on physiological or pathological conditions. Remarkably, IL-6 is highly expressed in COPD, and considered to be a biomarker of systemic inflammation, which is associated with both sarcopenia and bone loss. For osteokines, receptor activator of nuclear factor kappa-B ligand (RANKL), a classical regulator of bone metabolism, was recently found to play a critical role in skeletal muscle atrophy induced by chronic cigarette smoke (CS) exposure. In this focused review, we described evidence for myokines and osteokines in the pathogenesis of skeletal muscle dysfunction/sarcopenia and osteoporosis in COPD, and proposed muscle-bone crosstalk as an important mechanism underlying the coexistence of muscle and bone diseases in COPD.

## Introduction

Chronic obstructive pulmonary disease (COPD) is a prevalent chronic airway disease characterized by persistent airflow limitation and varying respiratory symptoms including chronic cough with or without sputum production, and exertional dyspnea which limits physical activity of the patient. The major risk factor for COPD is cigarette smoking, which leads to airway inflammation and alveolar destruction (emphysema), the pathological hallmark of the disease (1). COPD is now viewed as a systemic disease with significant comorbidities, such as sarcopenia, osteoporosis, cardiovascular diseases, lung cancer, gastroesophageal reflux, metabolic syndrome, anxiety and depression. These comorbidities contribute markedly to the mortality of COPD, and therefore their assessment and management are an integral part of COPD (1).

Skeletal muscle dysfunction/sarcopenia and osteoporosis are common in COPD, and their pathogenic mechanisms are believed to be associated with systemic inflammation (2). Muscle and bone are closely linked spatially. In addition to mechanical interactions, they also serve as endocrine organs to secrete myokines and osteokines to regulate bone metabolism and skeletal muscle growth and functions, respectively. In recent years, the role of muscle-bone crosstalk in the development of skeletal muscle dysfunction/sarcopenia or osteoporosis has attracted great attention (3), but its involvement in musculoskeletal comorbidities of COPD still awaits investigation. In this focused review, we searched for the studies of osteoporosis and skeletal muscle dysfunction/sarcopenia in COPD, and for muscle-bone crosstalk in general in the past 20 years. We described evidence for myokines and osteokines in the pathogenesis of skeletal muscle dysfunction/sarcopenia and osteoporosis in COPD, and put forward muscle-bone crosstalk as an important mechanism underlying the coexistence of muscle and bone diseases in COPD, aiming to promote research in this fields.

## Skeletal Muscle and Bone Comorbidities in COPD

Sarcopenia is an age-related progressive and systemic skeletal muscle disease associated with increased likelihood of adverse consequences such as falls, fractures, physical disability, hospitalizations and mortality (4). The European Working Group on Sarcopenia in Older People (EWGSOP) developed a practical clinical definition and diagnostic criteria for sarcopenia based on three criteria: muscle quantity or mass, muscle strength, and physical performance (4), which has been adopted by many studies. A meta-analysis reported that the overall pooled prevalence estimate of sarcopenia in people with COPD was 27.5% (95%CI, 18.4%–36.5%) (5), and the prevalence increased with age, degree of airflow limitation and severity of disease (6). Moreover, with the same degree of airway obstruction, sarcopenia is more prevalent in individuals with emphysema than in those with airway-type COPD (7). Skeletal muscle dysfunction occurs in patients with COPD and affects both respiratory and nonrespiratory muscles. Compared with upper extremities, skeletal muscle dysfunction is more significant in lower extremities (such as quadriceps femoris), which compromises the ambulatory capacity of COPD patients and has devastating effects on their daily lives (8). Until present, most studies on skeletal muscle dysfunctions in COPD have mainly focused on certain skeletal muscle groups, which often cannot accurately reflect the real prevalence and severity of the disease. For example, one study showed that there was no difference in the prevalence of sarcopenia between stable COPD patients with or without quadriceps weakness, and nearly 1/3 of those with sarcopenia had preserved quadriceps strength (6). Therefore, it is recommended that patients with COPD should be evaluated comprehensively based on the diagnostic criteria for sarcopenia. Currently, multiple studies have shown that sarcopenia/skeletal muscle dysfunction in COPD is associated with more severe airflow obstruction, emphysema, dyspnea score (modified British medical research council, mMRC), decreased quality of life and exercise capacity, frequent exacerbations and increased mortality (9–11). Exercise training is currently the most effective non-pharmacological intervention to improve skeletal muscle function in COPD. COPD patients with sarcopenia respond well to pulmonary rehabilitation, similar to those without sarcopenia (6), with improvement in exercise ability, upper and lower extremity strength, functional performance and health status.

Osteoporosis, a systemic skeletal disease characterized by low bone mineral density(BMD) and/or microarchitectural deterioration of bone, is another common comorbidity of COPD, which is associated with increased bone fragility/fracture, lower mobility and alteration in postural balance (12). A latest meta-analysis reported that the overall pooled prevalence of osteoporosis in patients with COPD was 38% (95%CI, 34%-43%), and BMI<18.5 kg/m^2^ and the presence of sarcopenia were significant risk factors for osteoporosis in COPD (13). Osteoporosis in COPD patients is usually asymptomatic, and often diagnosed until bone fractures occur, and therefore the prevalence of osteoporosis in COPD is often under-estimated. The risk of bone fracture depends on bone strength, which is comprised of BMD and bone quality, and BMD accounts for about 70% of bone strength (14). Due to the lack of precise evaluation of bone quality, the diagnosis of osteoporosis mainly depends on BMD, however, simply measuring BMD cannot fully reflect the risk of bone fracture. Thus, although BMD is decreased as Global Initiative for Chronic Obstructive Lung Disease(GOLD) stage advances in COPD patients, the prevalence of bone fracture appears to be independent of the GOLD stage, and the actual prevalence is higher than that predicted by BMD (15). It was reported that about 40% of patients with COPD suffered at least one event of vertebral fracture (16), with thoracic fracture as the most common  (17). Vertebral fracture can cause back pain, thoracic deformities, kyphosis and height loss, leading to impaired pulmonary function (18). In addition, low BMD/osteoporosis and related fractures are also associated with emphysema, frequent exacerbations (19), increased hospitalization rates and mortality (20), and decreased quality of life in patients with COPD (21).

## Muscle-Bone Crosstalk

Skeletal muscle dysfunction/sarcopenia can be caused by an imbalance of muscle protein synthesis and degradation, inflammation, mitochondrial dysfunction, myosatellite cell injury and disturbed calcium homeostasis (8, 22). There are many common risk factors between sarcopenia and osteoporosis in COPD, including systemic inflammation (such as enhanced tumor necrosis factor (TNF)-α and IL-6), cigarette smoking, hypoxemia and/or hypercapnia, malnutrition, oxidative stress and reduced level of physical activity (8, 23). Up to 70% of COPD patients with osteoporosis also show signs of muscle wasting (24), and sarcopenia is also an independent risk factor for osteoporosis in patients with COPD (13). Therefore, we hypothesize that there are interactions between muscle and bone comorbidities in COPD.

Skeletal muscle and bone are inextricably linked genetically, molecularly, and mechanically, and they are closely connected spatially (25). They have various functions in common, such as maintaining posture, locomotion, promoting breathing, protecting internal organs and coordinating overall energy consumption. Studies have also shown that muscle and bone can serve as endocrine organs in producing myokines and osteokines, respectively, which can interact within the so-called muscle-bone unit (26).

### Biomechanical Action of Muscle-Bone

There is a close mechanical relationship between skeletal muscle and bone, with the bone providing the muscle with a point of attachment and acting as a lever, and the muscle acting as a pulley to move the body (25). The coordination of muscle mass and bone mass is achieved by mechanical signals produced by muscle strength that transmits anabolic activity in adjacent bones (27). When the stimulus exceeds a certain threshold of mechanical strain, it can induce bone anabolism and enhance bone hardness, while preventing bone damage caused by increased mechanical load. Conversely, if the stimulus is below this threshold of mechanical strain, bone resorption will occur in related bone tissues (27). In daily activities, the mechanical relationship between muscle and bone occurs all the time, and exercise training is just one of the manifestations of the mechanical connection between muscle and bone. Exercise can produce mechanical signals through the physical connection between muscle and bone to affect bone remodeling. Although exercise training was generally considered to be beneficial for improving the loss of muscle mass and bone mass in most cases, some studies indicated that the role of exercise on improving bone loss was controversial. For example, studies showed that resistance training was associated with the improvement of femur bone mass (28), while endurance training might not improve bone mass (29). One study even showed that transient endurance exercise promoted bone resorption and stimulated upregulation of sclerostin, a bone formation inhibiting protein (30). From the biomechanical view, the decrease of muscle mass and function will lead to the reduction of mechanical load on the bone, and eventually lead to bone loss, which is consistent with the coexistence of sarcopenia and osteoporosis in many pathological conditions. However, the loss of bone mass cannot fully explain sarcopenia, nor does muscle atrophy fully explain osteoporosis (31). This suggests that there are other mechanisms of muscle-bone crosstalk besides biomechanical action.

### Biochemical Action of Muscle-Bone

Muscle and bone are two major components of the musculoskeletal system, and they are closely related in embryonic development, postnatal growth and development, and aging (31). In addition to their mechanical connection, the chemical crosstalk between muscle and bone has become a hot topic of research in recent years. Accumulating evidence indicates that muscle and bone can secrete cytokines to act on each other in autocrine, paracrine or endocrine manners. A variety of myokines and osteokines have been found to play critical roles in this muscle-bone crosstalk, including myokines such as myostatin, irisin, IL-6, and osteokines such as RANKL, OPG and osteocalcin.

#### Myokines

##### Myostatin

**Figure 1:**
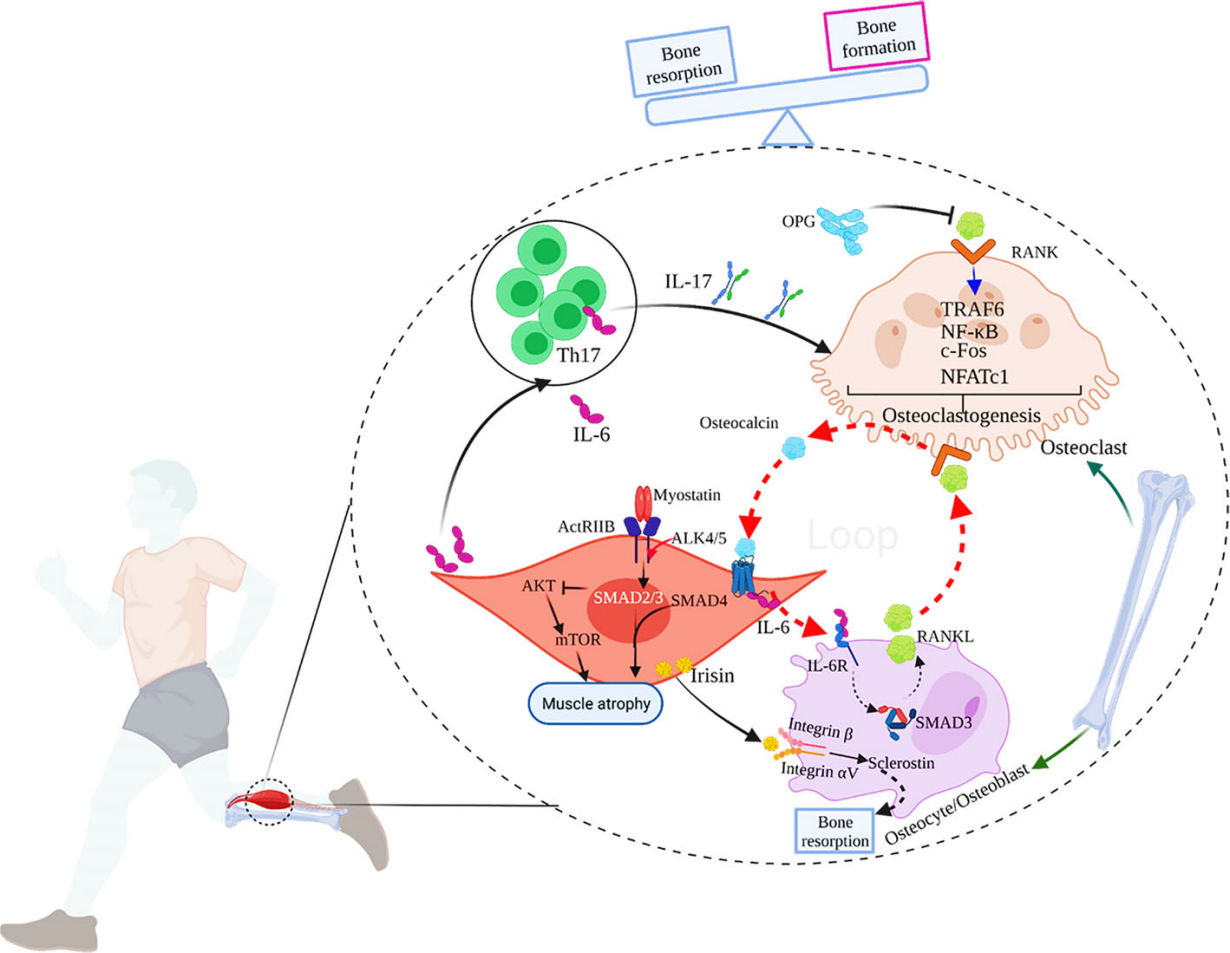
The main mechanisms of muscle-bone crosstalk. 1) IL-17 secreted by Th17 cells induces RANKL expression in mice, which combines with RANK on the surface of osteoclasts to activate TRAF6 and c-Fos to induce osteoclastogenesis, and eventually, bone resorption occurs. OPG, the decoy receptor of RANKL, can prevent RANKL from activating RANK in the extracellular environment, thereby inhibiting osteoclast formation and bone loss; 2) During exercise, the muscle-derived IL-6 (mIL-6) acts on the IL-6 receptor on the surface of osteoblasts and increases the production of RANKL, subsequently promoting osteoclast differentiation and the secretion of bioactive osteocalcin through RANKL/RANK pathway in osteoclasts. In turn, osteocalcin also enhances the production of mIL-6 during exercise. Thus, a feed-forward loop between muscle and bone favors exercise adaptations. 3) Myostatin signals through ActRIIB, which forms a heterodimer with ALK 4/5. The intracellular ALK4/5 phosphorylates SMAD2 and SMAD3, and then forms a complex with SMAD4. The complex translocates to the nucleus to regulate the transcription of genes involved in the proliferation and differentiation of skeletal muscle precursor cells. The activation of SMAD2 and 3 by myostatin also inhibits the Akt/mTOR pathway in response to pro-growth signals, and therefore, suppresses protein synthesis. Ultimately, myostatin contributes to muscle atrophy. 4) Irisin produced by skeletal muscles during exercise acts on integrin αV/β on the surface of osteocytes to promote sclerostin production, leading to bone resorption. 5) IL-6 can also promote the generation of IL-17.

Myostatin, also known as growth differentiation factor 8 (GDF-8), belongs to the transforming growth factor (TGF)-β superfamily. Myostatin can inhibit the proliferation and differentiation of myoblasts and promote muscle atrophy, and is a potent negative regulator of skeletal muscle growth and development (32). Myostatin signals through the activin type IIB receptor (ACVR2B or ActRIIB), which is widely expressed and forms a heterodimer with activin-like kinase 4 (ALK4, also known as ACVR1B) or ALK5 (also known as TGF-βR1) (33). The intracellular serine/threonine kinase domain of ALK4 and ALK5 phosphorylates SMAD Family Member 2 (Smad2) and Smad3, and then forms a complex with Smad4. The complex translocats to the nucleus to regulate the transcription of genes involved in the proliferation and differentiation of skeletal muscle precursor cells (34, 35), and in protein degradation pathways in mature myofibers (36). The activation of Smad2 and Smad3 by myostatin can also inhibit the Akt/mammalian target of rapamycin (mTOR) pathway in response to pro-growth signals, and therefore, suppresses protein synthesis (37) (**Figure 1**). Earlier studies also confirmed that human and animals with a mutation in the myostatin genes showed a remarkable growth in muscle mass. A decoy receptor (ACVR2B/Fc) was found to be a potent inhibitor of myostatin and as a result, could induce marked muscle growth when given systemically to wild type mice and microgravity-exposed mice respectively (38). Furthermore, studies showed that the expression of myostatin mRNA was downregulated in skeletal muscles after acute exercise and was even further decreased over long-term physical activity in sedentary men, and the expression of myostatin in muscles was associated with an impaired insulin sensitivity (39), suggesting that downregulation of myostatin in response to exercise may improve muscle performance.

Myostatin also promotes osteoclast development and inhibits bone formation (40). Myostatin alone cannot induce bone marrow-derived macrophages to form osteoclasts, but the presence of myostatin with classical signals [RANKL and macrophage colony-stimulating factor (M-CSF)] dramatically improved the ability of osteoclast precursors to differentiate into mature osteoclasts *in vitro* (40). On the gene expression level, myostatin can increase the nuclear translocation of Smad2-dependent nuclear factor of activated T cells, cytoplasmic 1 (NFATc1) and subsequently up-regulate the expression of osteoclast differentiation genes (40). The role of myostatin in osteoclast differentiation has also been confirmed in animal models. For instance, deficiency or pharmacological inhibition of myostatin prominently diminished osteoclast formation and promoted bone destruction in the human TNF-α transgenic (hTNFtg) mouse model of rheumatoid arthritis as well as in the serum-transfer-induced arthritis model (40). Furthermore, myostatin may also influence the expression of other cytokines in muscle-bone crosstalk. Knockout of myostatin genes in mice, for example, resulted in enhanced expression of irisin in muscles (41).

##### Irisin

**Figure 2:**
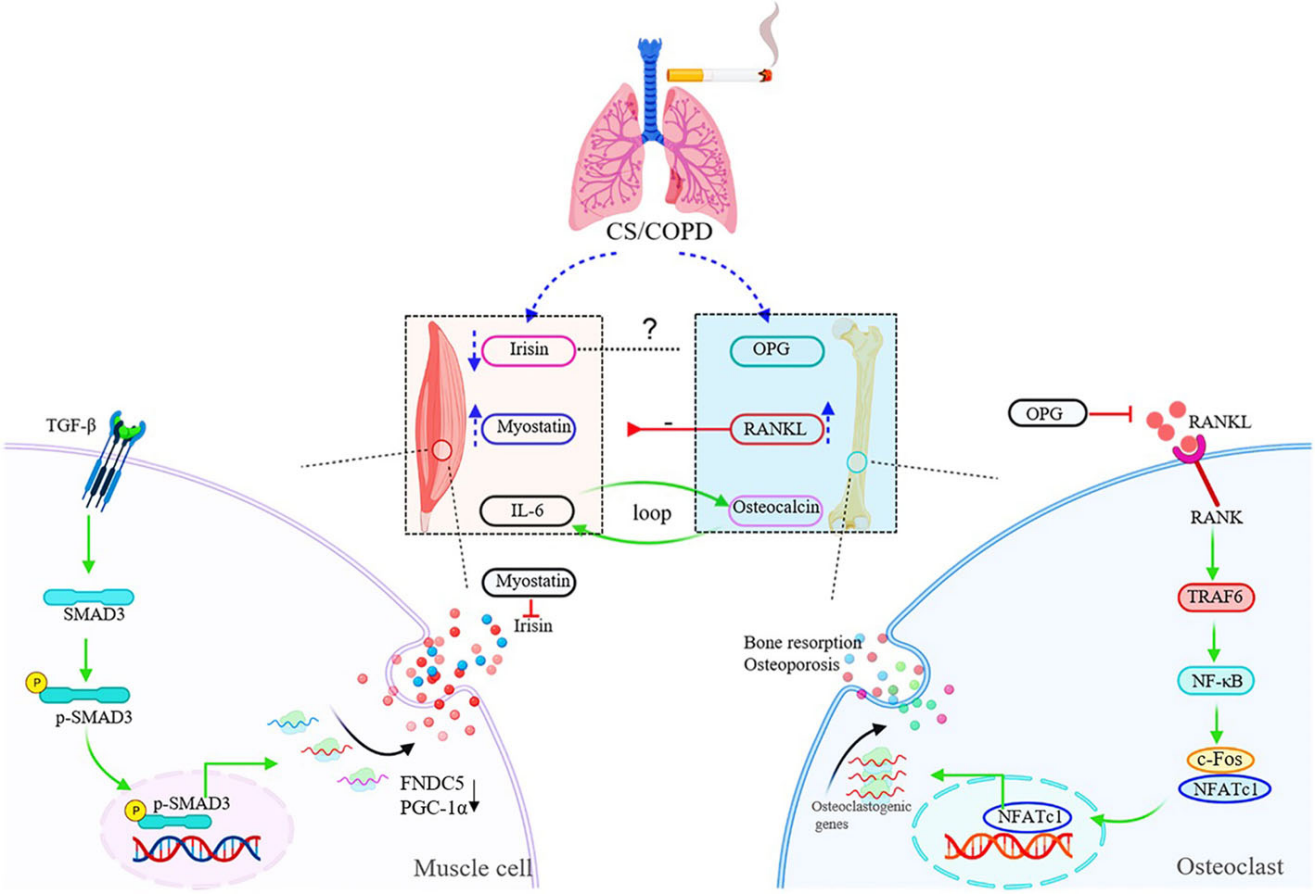
Schematic diagram of muscle-bone crosstalk in COPD. In COPD, the serum levels of irisin are decreased, while myostatin and RANKL are up-regulated in both circulation and skeletal muscles. The raised expression of RANKL induced by chronic cigarette smoke exposure contributes to muscle atrophy/skeletal muscle dysfunction through the RANKL/RANK pathway. The figure also shows the specific signaling pathway of irisin and RANKL. TGF-β binds to TGF-βR1/TGF-βR2 complex, and Smad3 is phosphorylated and translocated into the nucleus to bind the promoters of PGC-1α and FNDC5 to suppress their transcription, and suppression of FNDC5 transcription and protein leads to decrease in circulating irisin *in vitro*. RANKL activates the homologous receptor RANK on the surface of osteoclasts and osteoclast precursors, and activated RANK causes the recruitment of the adapter protein TRAF6, leading to NF-κB activation and translation of NF-κB to the nucleus. NF-κB increases the expression of c-Fos, and c-Fos interacts with NFATc1 to trigger the transcription of osteoclastogenic genes.

Irisin is a newly discovered myokine, mainly derived from skeletal muscles (42). Irisin is proteolytically cleaved from the fibronectin type III domain-containing protein 5 (FNDC5) gene products and secreted into the blood (42). Irisin is regulated by exercise, and its plasma concentration increases in a dose-dependent manner with increase in aerobic exercise intensity (43). In a meta-analysis, Fox et al. evaluated the effect of an acute bout of exercise (including aerobic and/or resistance exercise) on circulating irisin levels, and found that the circulating irisin levels boosted by 15% (95%CI: 10.8%-19.3%) after exercise compared to baseline (44). The expression of irisin is regulated by peroxisome proliferator-activated receptor γ-coactivator 1α (PGC-1α) and Smad3. In contrast to wild type mice, Smad3^-/-^ mice showed higher serum and skeletal muscle FNDC5 protein levels with the same exercise protocol (45). TGF-β binds to TGF-βR1/TGF-βR2 complex, and Smad3 is phosphorylated and translocated into the nucleus to bind the promoters of PGC-1α and FNDC5 to suppress their transcription, and suppression of FNDC5 transcription and protein leads to decrease in circulating irisin *in vitro* (45) (**Figure 2**).

Irisin plays a positive role in regulating muscle mass by promoting differentiation of myoblasts (46). In last few years, several important studies have found that irisin was also a pivotal regulator of bone metabolism. Colaianni et al. found that male C57BL/6 mice injected with recombinant irisin (100μg/kg, once a week) for a month showed increased cortical tissue BMD of femora and tibia, accompanied by increased osteoblast numbers (47). Irisin promoted the differentiation of bone marrow stem cell-derived osteoblasts *in vitro* (47). Up till now, most studies support the role of irisin in promoting bone formation, such as in inflammatory bowel disease, hind-limb unloading, and microgravity (weightlessness) induced bone loss models, and exogenous administration of recombinant irisin alleviated bone loss (48–50). Nevertheless, a more recent study, firstly identified integrin αV/β5 as the receptor of irisin, found that genetic deletion of FNDC5/irisin completely blocked trabecular bone loss in mouse models of osteoporosis caused by ovariectomy (51). Irisin induced up-regulated expression of sclerostin and receptor activator of nuclear factor kappa-B ligand (RANKL) through integrin αV/β5 in the osteocyte membrane, thereby promoting bone resorption (51) (**Figure 1**). This discovery prompted discussion to re-examine the role of irisin in muscle-bone crosstalk (52).

#### Interleukin 6 (IL-6)

IL-6 is a classical pro-inflammatory cytokine mainly produced by T lymphocytes and macrophages. Skeletal muscles can also produce a large amount of IL-6 in response to muscle contraction, which is released into blood stream during exercise, and therefore IL-6 is also regarded as a myokine. In skeletal muscles, IL-6 acts as a pleiotropic factor and appears to have at least two different conflicting functions (53). In exercise and physiological conditions, IL-6 produced by skeletal muscles is increased for several hours in the circulation, which promotes the activation of the signal transducer and activator of transcription 3-suppressor of cytokine signaling 3 (Stat3-SOCS3) signaling pathway through autocrine effects and plays a positive role in regulating the myogenic differentiation (54, 55). However, in pathological conditions, including muscular dystrophy and chronic inflammatory diseases, there is a chronic and lasting elevation of the circulating IL-6. Although Stat3-SOCS3 signaling is also activated in the target skeletal muscle under these pathological conditions, the autocrine loop of regulation in the action of IL-6 is totally lost (53). Besides, IL-6 combined with other molecules negatively regulates muscle differentiation and promotes muscle atrophy (53). Although IL-6 is a well-known pro-inflammatory cytokine, it can also exert anti-inflammatory effects under certain conditions. For instance, the exercise-induced rise in IL-6 promoted production of IL-1 receptor antagonist (IL-1ra) and IL-10 (54), which stimulated an anti-inflammatory systemic environment during exercise. A recent study confirmed through genetic means that the vast majority of circulating IL-6 detectable during exercise originated from muscles (56), and the magnitude of exercise-induced elevation of plasma IL-6 depended on exercise mode, intensity and duration, as well as energy availability (54, 57). Furthermore, it’s reported that the amount of regular physical activity was negatively associated with the basal circulating IL-6 levels (54), that is to say, the less physical activity, the higher basal plasma IL-6. This regulation was attributed to the effects of training adaptations. However, it also has been demonstrated that acute exercise upregulated IL-6 receptor (IL-6R) gene expression in skeletal muscles, and long-term training increased basal IL-6R mRNA levels (57). Therefore, the downregulation of systemic levels of IL-6 induced by exercise training may be compensated for the enhanced production of IL-6R, suggesting a sensitization of skeletal muscles to IL-6 at rest. Meanwhile, it is worth noting that IL-6 signals in osteoblasts to promote cell differentiation and the release of bioactive osteocalcin into circulation, and the muscle-derived IL-6 (mIL-6) promotes nutrient uptake and catabolism in myofibers during exercise in an osteocalcin-dependent manner, thus enhancing exercise capacity (56). In addition to regulating muscle growth and enhancing exercise capacity, IL-6 appears to induce bone resorption through RANKL-dependent enhanced osteoclastogenesis/osteoblast differentiation (58) (**Figure 1**) as well as *via* osteoblast-derived prostaglandia E2 (PGE2)-dependent osteoclast activation (59, 60).

#### Osteokines

##### RANKL and Osteoprotegerin(OPG)

RANKL is produced by osteoblasts, stromal cells, and other types of cells under the regulation of various hormones and cytokines. Osteoblasts and stromal cells also produce OPG-the decoy receptor of RANKL. Both RANKL and OPG belong to the TNF family. RANKL activates the homologous receptor RANK on the surface of osteoclasts and osteoclast precursors, and activated RANK causes the recruitment of the adapter protein TNF receptor associated factor 6 (TRAF6), leading to NF-κB activation and translation of NF-κB to the nucleus. NF-κB increases the expression of c-Fos and c-Fos interacts with NFATc1 to trigger the transcription of osteoclastogenic genes (61), which contributes to bone resorption and osteoporosis. OPG can prevent RANKL from activating RANK in the extracellular environment, thereby inhibiting osteoclast formation and bone loss (61) (**Figure 2**). In wild-type mouse models, RANKL and RANK are expressed in both bones and muscles, and RANKL is particularly highly expressed in oxidative muscles like the soleus dominated by type I fibers (62). In huRANKL transgenic mice, the overexpression of huRANKL not only induced bone loss, but also resulted in decreased leg muscle, lower maximal speed and limb force, decreased number of both type I and II myofibers, and impaired glucose metabolism (62). These changes were associated with the increased levels of antimyogenic and inflammatory gene expression in muscles, such as myostatin and protein tyrosine phosphatase receptor-γ (62). In mouse models, intervention with OPG-Fc (an inhibitor of both RANKL and TNF-related apoptosis-inducing ligand) and denosumab (a selective RANKL inhibitor) rescued muscle weakness to the similar levels, suggesting that the effect of RANKL on muscle is mainly mediated by RANK signals (62). In postmenopausal women, the elevated expression of RANKL plays a pivotal role in the development of osteoporosis (63). Denosumab treatment not only improved BMD and reduced the risk of fracture, but also, interestingly, increased the appendicular lean mass and grip strength (62, 64).

##### Osteocalcin

Osteocalcin is a small γ-carboxyglutamate protein expressed by osteoblasts, and is only expressed in the late differentiation stage of osteoblasts and after the arrest of proliferation, under the regulation of Runx2/Cbfa1 transcription factor (65). γ-carboxylation occurs on three glutamate residues of osteocalcin before osteocalcin is secreted by osteoblasts. The post-translational modification increases the affinity of osteocalcin to hydroxyapatite. Therefore, the majority of the osteocalcin secreted by osteoblasts is stored in bone matrix, which constitutes the most abundant non-collagenous polypeptides (66). Osteocalcin in circulation exists in two forms: γ-carboxylated osteocalcin and uncarboxylated osteocalcin, and only uncarboxylated osteocalcin plays the role of endocrine hormone (67). In the process of bone resorption under acidic conditions, osteoclasts decarboxylate γ-carboxylated osteocalcin in bone matrix, activate and release bioactive osteocalcin into blood (68). The effects of osteocalcin on bone metabolism remain controversial from studies *in vivo* and *in vitro*. It is speculated that osteocalcin plays a dual role. On the one hand, osteocalcin is a biochemical marker of bone turnover and can regulate bone remodeling by regulating the activity of osteoblasts and osteoclasts. On the other hand, it is a regulator of bone mineralization (69).

Previous studies explored the role of osteocalcin in regulating glucose homeostasis, insulin sensitivity and energy metabolism (68, 70). In recent years, more studies have found that osteocalcin plays an important role in regulating muscle quantity, muscle function, and movement adaptability (71). It has been identified that G-protein coupled receptor C family 6a (Gprc6a) is the osteocalcin receptor on cells, which is highly expressed in skeletal muscles, especially in the oxidative muscles that require long-term work, such as the soleus muscle (71). Uncarboxylated osteocalcin could promote myoblast proliferation through the PI3K/Akt/p38 MAPK pathway, or through the Gprc6a-ERK1/2 signaling pathway *in vitro* (72). Mera et al. demonstrated a notable decrease in muscle mass in Gprc6a-deficient mice, and the administration of osteocalcin ameliorated muscle mass in wild type 9-month-old mice (73), suggesting that osteocalcin signaling in myofibers is necessary for maintaining muscle quantity in elderly mice, partly due to the promotion of protein synthesis in the myotubes. In early adulthood, circulating osteocalcin levels begin to decline sharply, especially during aging; Circulating osteocalcin levels in women and men reach their lowest levels before the age of 30 and 50, respectively (71). Interestingly, exercise has been shown to increase circulating osteocalcin levels. For example, a bout endurance aerobic-based exercise in 3-month-old mice (74) and a 45-minute exercise in young women (71) significantly increased circulating osteocalcin levels. Osteocalcin not only favors the uptake and catabolism of glucose and fatty acids in myofibers, but also enhances the production of mIL-6 during exercise, a myokine that promotes the secretion of bioactive osteocalcin (**Figure 1**), while exogenous osteocalcin administration improved the exercise capacity of mice (71). Hence, it’s possible that osteocalcin signaling in myofibers may be a novel and promising means to attenuate the age-related decline in muscle function.

#### Other Cytokines

In addition to the molecules described above, muscle and bone also produce other myokines and osteokines (75, 76), such as insulin-like growth factor (IGF)-1, fibroblast growth factor (FGF)-2, PGE2, IL-7, IL-15, beta-aminoisobutyric acid (BAIBA), TGF-β, and sclerostin, which form a delicate network in muscle-bone crosstalk. Whether one or more of these molecules are involved in musculoskeletal comorbidities of COPD still needs further investigation, so we will not introduce these cytokines in more details here. We mostly focused on those involved in bone and muscle comorbidities in COPD, and those believed to play key roles in muscle-bone crosstalk.

## Muscle-Bone Crosstalk in COPD

Aging, chronic inflammation, inactivity, bed rest and other pathological conditions in COPD result in the loss of muscle and bone mass and functional impairment. Although the clinical significance of sarcopenia and osteoporosis is well recognized, the molecular mechanisms under these pathological conditions remain to be investigated. However, myokines and osteokines potentially involved in muscle-bone crosstalk in COPD have newly been revealed, including IL-6, irisin, myostatin, RANKL, osteocalcin, etc.

It is well known that COPD is characterized by both local and systemic inflammation, and IL-6, as a key pro-inflammatory cytokine, is highly expressed in patients with COPD. Elevated circulating IL-6 levels are associated with decreased forced expiratory volume in one second (FEV1), strength of quadriceps femoris and exercise capacity in COPD patients (77). In a 3-year study of inflammatory markers in patients with COPD, elevated serum IL-6 was found to predict increased mortality (78). However, as described in the previous section, IL-6 is also a myokine that plays both anti-inflammatory and pro-inflammatory roles in skeletal muscles. It can regulate the differentiation of osteoclasts and enhance exercise adaptability. From the perspective of muscle-bone crosstalk, the role of IL-6 in COPD should be re-examined.

Some studies have examined the expression of irisin in COPD, and its association with emphysema and exercise. Compared with healthy controls, COPD patients showed decreased level of serum irisin which was positively correlated with physical activity (79). Exercise training raised circulating irisin in a chronic cigarette smoke (CS)-exposure induced mouse model of COPD (80). In patients with COPD, serum irisin levels were significantly correlated with diffusing capacity of lung for carbon monoxide/alveolar volume (DL_CO_/VA) and percentage of low-attenuation area (LAA%) (81). Interestingly, an experimental study showed that irisin ameliorated emphysema in a CS-induced model of COPD in mice, in which irisin played an anti-oxidative role through nuclear factor erythroid 2-related factor 2 (Nrf2, a transcription factor with antioxidant properties) and heme oxygenase-1 (HO-1, an antioxidant in the antioxidant pathway) (80). *In vitro*, irisin was found to promote the expression of Nrf2 and decrease apoptosis induced by CS exposure in A549 cells (81). These findings suggest that irisin, in addition to its role as a myokine, may be also involved in the pathogenesis of emphysema induced by epithelial apoptosis in COPD.

Myostatin is a negative regulator of skeletal muscle growth and development, and some studies have reported dysregulated expression of myostatin in COPD and its association with skeletal muscle dysfunction. Serum myostatin levels were elevated, and negatively correlated with total muscle mass in male COPD patients (82), and myostatin mRNA expression was also elevated in skeletal muscles from COPD patients (83). Myostatin maintains the satellite cells (SCs) in quiescent state, and its absence can trigger the activation of SCs (84). In addition, inhibition of myostatin alleviated muscle atrophy by upregulating muscle regeneration markers in limb muscles of rats (85). A recent study found that the markers of muscle regeneration (Pax-7, Myf-5, MyoD and myogenia) and the numbers of Pax-7+/Myf-5- SCs in the vastus lateralis of sarcopenic COPD patients were decreased, while markers of muscle injury and the myostatin level were increased (86). In a clinical study of sarcopenic COPD patients, Bimagrumab (an ACVR2 inhibitor that blocks the myostatin pathway) was found to safely increase skeletal muscle mass in patients with COPD, but no improvement in muscle function or physical performance was observed (87). Clinically, pulmonary rehabilitation is the most effective non-pharmacological treatment for COPD patients at present. Pulmonary rehabilitation was found to improve quadriceps femoris strength and physical performance (88) and decrease myostatin expression in quadriceps femoris (89). Moreover, resistance training can not only attenuate the expression of myostatin in skeletal muscles, but also up-regulate the proportion of myogenin/MyoD, and safely and effectively counteract skeletal muscle dysfunction in acute exacerbation of COPD (90).

Latest studies also indicate that osteokines play critical roles in muscle-bone crosstalk in COPD. BMD was found to be associated with the imbalance of RANKL/OPG and systemic inflammation in peripheral blood of patients with COPD (91). In addition, in IL-17A knockout mice, bone loss induced by chronic CS exposure was attenuated, with downregulation of RANKL, suggesting that IL-17 promoted bone resorption by inducing RANKL expression in this model (92). Our most recent study found that chronic CS exposure induced skeletal muscle atrophy and muscle weakness in mice, with up-regulated expression of RANKL and its receptor RANK in skeletal muscles. RANKL neutrolization attenuated skeletal muscle dysfunction and reduced the expression of myostatin and MuFR1/Atrogin1. *In vitro*, CS extract induced up-regulation of RANKL/RANK in skeletal muscle cells, and blocking RANKL down-regulated myostatin expression (93). These results indicate that the RANKL/RANK pathway plays a key role in skeletal muscle atrophy induced by chronic CS exposure.

To our knowledge, there are few studies on osteocalcin in COPD, and it is only used as a marker of bone turnover or bone formation (94, 95), without further investigation on the mechanism of osteocalcin in the muscle-bone crosstalk of COPD. As mentioned earlier, osteocalcin does play a role in muscle-bone crosstalk during exercise. During exercise, a feedback loop between bone (via osteocalcin) and muscle (*via* IL-6) enhances exercise adaptation: osteocalcin promotes nutrient absorption and catabolism of myofibers, and osteocalcin can trigger IL-6 expression and secretion in skeletal muscles. IL-6 also enhances the production of bioactive osteocalcin (56). The roles of osteocalcin and IL-6 in muscle-bone comorbidities of COPD deserve further investigation. Based on the evidence available to date, we propose a muscle-bone crosstalk mechanism for skeletal muscle dysfunction/sarcopenia and osteoporosis in COPD, as shown in **Figure 2**.

## Conclusion

COPD is a major cause of chronic morbidity and mortality worldwide, with many people dying prematurely from the disease or its complications. Sarcopenia and osteoporosis, as important comorbidities of COPD, are associated with COPD severity and prognosis. Currently, the mechanisms underlying the muscle-bone relationship in COPD is still unclear, and there is still a lack of comprehensive and systematic measures to improve the condition and prognosis of COPD and its comorbidities in clinical practice. Fortunately, studies have revealed the potential role of muscle-bone crosstalk involving myokines and osteokines, such as IL-6, irisin, myostatin, RANKL/RANK and osteocalcin, in the development of sarcopenia and osteoporosis in COPD. Identification of key molecules in the pathogenesis of musculoskeletal diseases holds promise for precision therapy of comorbidities in COPD. Importantly, rehabilitation is now the most effective non-pharmacological therapy for improving outcomes of COPD (1), but its impact on the delicate network of myokines and osteokines is underappreciated. Further understanding of muscle-bone crosstalk in physical exercise in COPD may shed light on implementation of better modalities of non-pharcological management for a disease currently without a pharmalogical cure.

## Author Contributions

YS conceived and revised this manuscript. LZ collected materials, wrote and revised the manuscript. All authors contributed to the article and approved the submitted version.

## Funding

This work was supported by the National Natural Science Foundation of China (81770040, 81970041) and Natural Science Foundation of Beijing Municipality (7192224).

## Conflict of Interest

The authors declare that the research was conducted in the absence of any commercial or financial relationships that could be construed as a potential conflict of interest.

## Publisher’s Note

All claims expressed in this article are solely those of the authors and do not necessarily represent those of their affiliated organizations, or those of the publisher, the editors and the reviewers. Any product that may be evaluated in this article, or claim that may be made by its manufacturer, is not guaranteed or endorsed by the publisher.
